# Ambient Air Deposition Allows Reaching Record Light Use Efficiency in FAPbI_3_ Perovskite Solar Cells

**DOI:** 10.1002/advs.202501533

**Published:** 2025-05-08

**Authors:** Nadir Vanni, Mario Calora, Lucia Mercurio, Antonella Giuri, Anna Paola Caricato, Veronica Chierchia, Claudio Carati, Riccardo Po', Paolo Biagini, Salvatore Valastro, Emanuele Smecca, Giovanni Mannino, Alessandra Alberti, Aurora Rizzo

**Affiliations:** ^1^ Dipartimento di Matematica e Fisica “E. De Giorgi” Università del Salento Campus Ecotekne via Arnesano Lecce 73100 Italy; ^2^ CNR NANOTEC–Istituto di Nanotecnologia c/o Campus Ecotekne Via Monteroni Lecce 73100 Italy; ^3^ New Energies, Renewable Energies and Materials Science Research Center Istituto Guido Donegani Eni S.p.A. via Fauser 4 Novara I‐28100 Italy; ^4^ CNR‐IMM Zona Industriale Strada VIII, 5 Catania 95121 Italy

**Keywords:** ambient air deposition, light use efficiency, perovskite solar cells, semitransparent

## Abstract

Semi‐transparent solar cells represent an exciting opportunity for sustainable energy production, thanks to the possibility of being integrated into buildings and urban environments, effectively exploiting the already existing space. Perovskite solar cells (PCSs) are ideal candidates, offering high power conversion efficiencies (PCEs) combined with a tuneable band gap and adjustable thickness, which allow a convenient modulation of the average visible transmittance (AVT). However, balancing high PCE and high AVT is a challenging target. This study uncovers that depositing the perovskite layer based on Formamidinium Lead Iodide (FAPbI_3_) thin films in ambient air, rather than in a nitrogen‐controlled atmosphere, allows an increase in the AVT value of up to > 40% while also enhancing the photovoltaic performance and stability. The optoelectronic quality of the as‐obtained perovskite layer is substantially enhanced, showing fewer defects and a superior morphology. As a result, the air‐deposited devices exhibit higher efficiency, which, combines with the enhanced AVT, results in a champion device with a light use efficiency (LUE) of 4.2%, having a PCE of 13.8% and AVT of 30.4%. The record LUE value and the possibility of being deposited in ambient air conditions pave the avenue toward the real‐world application of semi‐transparent PSCs.

## Graphical abstract

Ambient air deposition of Formamidinium Lead Iodide (FAPbI_3_) perovskite enables an increase in average visible transmittance (AVT) value by up to 40%, while also enhance the optoelectronic quality of the film, showing fewer defects and a superior morphology. As a result, semi‐transparent devices achieve a record light use efficiency (LUE) value of 4.2%, opening the way to the real‐world application of semi‐transparent perovskite solar cells.

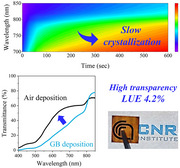

## 1 Introduction

In the forthcoming energy transition toward clean and inexhaustible energy sources, the search for new ways to produce green energy is deemed necessary. In this scenario, semi‐transparent photovoltaics (ST‐PVs) represent a highly desirable technological prospect as they can be integrated into windows of buildings or skyscrapers,^[^ ^1^ ^]^ thus increasing on‐site energy production and complementing conventional solar panels. This will maximize space for sunlight harvesting and minimize electrical transmission losses and the need for storage capacity by being closer to the source of use.^[^ ^2^ ^]^ Building‐integrated PV can also provide shade and regulate the generated heat in the building by partially absorbing and reflecting the incident light. Another interesting application for ST‐PVs can be found in agriculture, thanks to the possibility of obtaining tinted glass.^[^ ^3^, ^4^ ^]^

Among all of the technologies, perovskite solar cells (PSCs) are extremely attractive for ST‐PVs thanks to the high‐power conversion efficiency (PCE)^[^ ^5^ ^]^ combined with the possibility to easily tune the thickness and band gap^[^ ^6^ ^]^ of the active layer, thus the transparency.^[^ ^7^ ^]^ The exceptional optical and electrical properties, such as high optical absorption coefficient, high defect tolerance, and high carrier mobility,^[^ ^8^, ^9^, ^10^ ^]^ combined with the mild temperature and ease of deposition, e.g. solution coating methods, make it attractive to the photovoltaic industry.

To define the capacity of the device to transmit visible light, the first parameter is the average visible transmittance (AVT), whose values range between 0% and 50% for tinted non‐wavelength‐selective devices and values above 50% up to 90% for wavelength‐selective devices.^[^ ^11^ ^]^ However, since absorption and transmittance are competitive processes, achieving high efficiencies alongside high AVT values is challenging, therefore, the light use efficiency (LUE), defined as the product PCE × AVT, is another parameter often used to classify the efficiency of a semi‐transparent device. High AVT values for PSCs are usually achieved by reducing the thickness of the active layer^[^ ^12^, ^13^, ^14^ ^]^ or by incorporating mixed halide in the perovskite composition, which increases the band gap and the transparency at longer wavelengths but results in severe phase segregation processes and degradation.^[^ ^15^, ^16^ ^]^ 2D perovskites and additives have also been explored to control the crystallization and to improve the AVT and LUE values of semi‐transparent solar cells. Zou et al.^[^ ^17^ ^]^ achieved an LUE of 3.1% by blending 2D crystals with 3D Cs_0.13_FA_0.87_Pb(I_0.87_Br_0.13_)_3_ crystals, which provided a crystallization template and consequently enlarged the perovskite grains. Bisconti et al.^[^ ^18^ ^]^ used cellulose polymer to increase the AVT of MAPbI₃ by 44% while also enhancing the thermal stability of the PSC.

Herein it was found that depositing the active layer of Formamidinium Lead Iodide (FAPbI_3_) perovskite in ambient air results in an increase of up to >40% in AVT if compared to the sample fabricated in the controlled N_2_‐filled glovebox. This simple method allows for higher transparency without significantly lowering the thickness. Choosing FAPbI_3_ as the active layer helps to avoid the phase segregation issue completely, which can be encountered in mixed halide composition. The slightly lower thickness of the air‐deposited FAPbI_3_ combined with enlarged and more compact grains helps to obtain AVT values spanning from 25% to 42% in the range 400–800 nm by simply modifying the molarity of the perovskite's precursor solution. Importantly, the deposition in ambient air leads to a perovskite thin film with higher optical quality featuring enhanced photoluminescence lifetimes and reduced defect density. For those reasons, the ST‐PSCs fabricated in ambient air show higher power conversion efficiency, albeit with much improved AVT, if compared to the glovebox fabricated devices, with especially higher open circuit voltage (Voc) values as well as drastically enhanced stability. Combining the ideal band gap for sunlight harvesting of the FAPbI_3_ of 1.48 eV^[^ ^19^ ^]^ and the superior quality of the active layer with an enhanced AVT allows finding a balance between transparency and efficiency, eventually reaching a maximum LUE of 4.2%, which is a record value for FAPbI_3_ perovskite.^[^ ^20^ ^]^

## 2 Results

Perovskite materials are inherently unstable when exposed to moisture, as such active layer for PSCs is typically deposited in a controlled nitrogen‐filled glovebox environment.^[^ ^21^ ^]^ Nonetheless, moving toward ambient air deposition of perovskite is highly desirable to simplify the production and to reduce the costs of PSC, thus, speeding up the larger scale application and the future commercialization of the technology.^[^ ^22^, ^23^ ^]^ Given the perovskite sensitivity to water and oxygen, obviously, significant changes are found when the deposition is conducted in ambient air, as opposed to a controlled environment.^[^ ^24^, ^25^ ^]^

**Figure 1:**
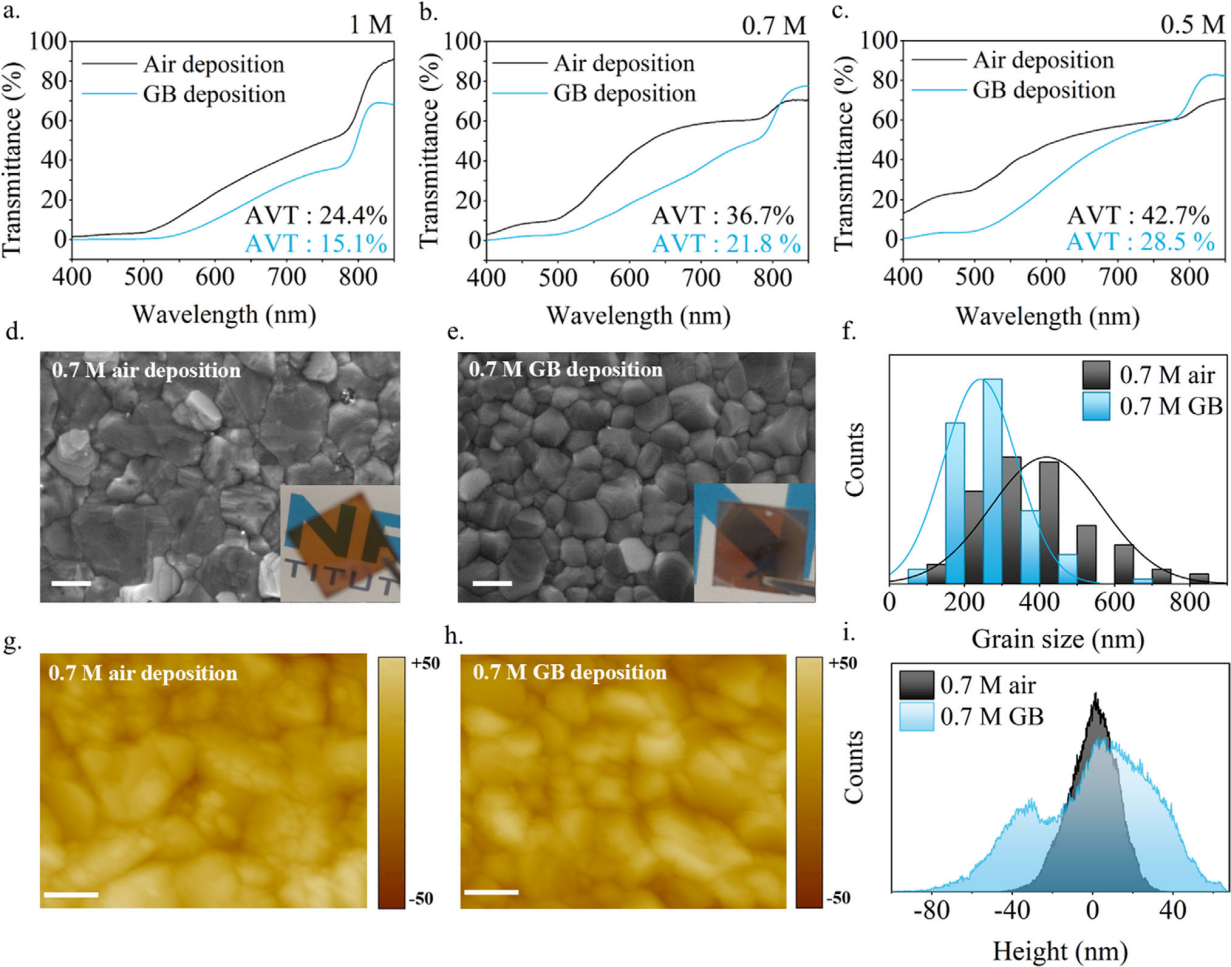
Transmittance spectra of FAPbI_3_ perovskite films on glass substrate deposited in the glovebox and in ambient air with a) 1 m concentration, b) 0.7 m concentration, c) 0.5 m concentration d) SEM image (scale 300 nm) of FAPbI_3_ 0.7 m deposited in the air e) SEM image (scale 300 nm) of FAPbI_3_ 0.7 m deposited in GB f) Grain size distribution of FAPbI_3_ deposited in ambient air and GB g) AFM image (scale 500 nm) of FAPbI_3_ 0.7 m deposited in the air h) AFM image (scale 500 nm) of FAPbI_3_ 0.7 m deposited in GB i) Height distribution of FAPbI_3_ deposited in ambient air and glovebox.

Herein, it was found that the ambient air‐deposited FAPbI_3_ films show a paramount enhancement of the transmittance. This phenomenon was initially investigated by depositing FAPbI_3_ films with decreasing concentrations in the glovebox (GB) and in ambient air with relatively high humidity in the 40%–60% range, where no differences were found. Three different concentrations were tested and referred to as 1, 0.7, and 0.5 m. The comparison of the transmittance spectra relative to the as‐prepared films is shown in **Figure** **1**a–c. Large area films (16 cm^2^) were also fabricated of the 1 m concentration and shown in the Supporting Information (Figure S1, Supporting Information). The transmittance spectra and calculated AVT values demonstrate that the FAPbI_3_ films deposited in air exhibit enhanced transparency across all investigated concentrations, with an observed increase in AVT value of up to > 40%. (Table S1, Supporting Information). The same effect was observed also in samples of FAPbI_3_ 1 m without methylammonium chloride (MACl) used as an additive in the formulation, in order to exclude MACl influence in the obtainment of perovskite films with higher transmittance (Figure S2, Supporting Information). The thickness of the samples was measured by both ellipsometry and profilometry and the samples deposited in air were found to be thinner but with a difference of less than 20% in thickness with respect to the same formulations deposited in the glovebox. This underlines a non‐linear correlation between the reduced thickness with the transmittance increase. The 0.7 m formulation deposited in air yielded in FAPbI_3_ films with a remarkable increase in AVT of 41% with respect to the glovebox‐deposited sample, with only a 16% difference in thicknesses (Table S1, Supporting Information), highlighting the effectiveness of the deposition in air in enhancing the optical properties of the perovskite without significantly affecting the film thickness. The thickness values were also confirmed by cross‐sectional Scanning Electron Microscopy (SEM) images reported in Figure S3 (Supporting Information). Looking at the morphologies investigated through SEM in Figure 1d,e for the 0.7 m concentration and in Figure S4a,b (Supporting Information) for the other concentrations it is clear that the grains formed during the deposition in ambient air are enlarged if compared to the grains formed in the controlled environment. A more quantitative comparison is shown in Figures 1f and S4c (Supporting Information), where the grain size distribution and average values extracted via the image‐J program are reported. Moreover, Atomic Force Microscopy was performed on the 0.7 m samples to analyze the surface morphology (Figure 1g,h), showing lower roughness values for the 0.7 m air‐deposited sample, with surface roughness (Sq) of 11.91 nm compared to 29.03 nm of the GB sample, and a narrower height distribution shown in Figure 1i, further reducing the scattering effect on the perovskite surface.

**Figure 2:**
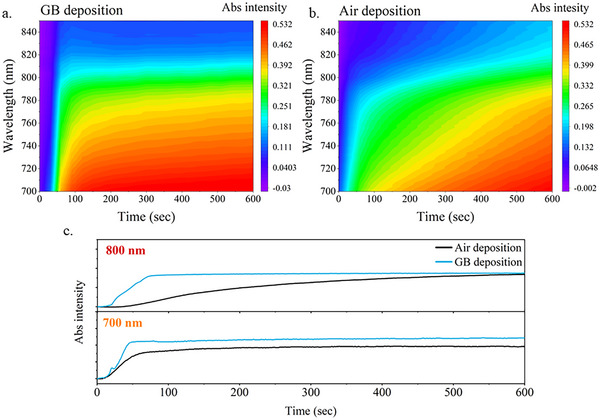
Time‐resolved absorption intensity maps during annealing of a) FAPbI_3_ 1 m sample deposited in glove‐box b) FAPbI_3_ 1 m sample deposited in ambient air c) Time‐evolution of absorbance at 800 and 700 nm during the annealing process.

To get an insight into the changes in the crystallization dynamics, absorbance spectra measurements during the annealing process of the perovskite films deposited in ambient air and in the glovebox were performed.^[^ ^26^ ^]^ Figure S5 (Supporting Information) shows the temporal evolution of the absorption spectrum of the 1 m samples deposited in ambient air and glovebox. When comparing the two deposition methods, the perovskite formation results accelerated in the case of glovebox deposition, reaching the maximum absorbance just after 1 min. This is especially true for the region from 850 to 700 nm, which is where the perovskite onset falls. In the absorption intensity maps for this region, shown in **Figure** **2**a,b, it is clearly visible how for the ambient air deposition the increase in absorption intensity is more gradual and stretched for several minutes, while for the glovebox deposition, the increase in intensity stops almost instantly and remains stable.

These dynamics were also confirmed by the evolution of absorbance taken at 800 and 700 nm, shown in Figure 2c. At both wavelengths, the glovebox‐deposited sample exhibits a faster kinetics with a sharp onset, indicating a fast crystallization, while for the air‐deposited sample, the onset is significantly wider. The slower crystallization induced in the air‐deposited sample promotes the increase in grain size seen in the SEM images and it is often associated with the formation of fewer defects and, in general, higher quality of the sample.^[^ ^27^, ^28^, ^29^ ^]^ A reasonable explanation of why the crystallization is slowed down can be given considering that the water present in the ambient moisture can partially solvate organic ammonium salts, such as formamidinium in this case, thus enhancing the mobility of the cation and encouraging the redissolution of smaller grains and promoting the formation of larger ones, consequently retarding the crystallization process. This aligns with previous reports that link the presence of water from ambient moisture during deposition to enlarged grain sizes in the perovskite films.^[^ ^30^, ^31^, ^32^ ^]^ By enhancing the grain dimensions and reducing grain boundaries, light scattering within the film is significantly minimized and the transparency is enhanced.^[^ ^33^, ^34^ ^]^ It is worth noting that it was verified that it is the deposition process that induces the observed changes, rather than the annealing process, as it was tested that the glovebox‐deposited sample does not change in terms of transmittance whether it is annealed in the glovebox or ambient air (Figure S6, Supporting Information).

The X‐ray diffraction (XRD) patterns are reported in Figure S7 (Supporting Information), in which the main diffraction peaks of the FAPbI_3_ α‐phase are individuated at 13.95°, 28.10° and 31.50°^[^ ^35^, ^36^ ^]^ together with some traces of PbI_2_, i.e., the peak of the at 12.6°.^[^ ^37^ ^]^ Those latter traces are present in all of the samples, nonetheless, it has been demonstrated in previous work that this is not detrimental to the device performance.^[^ ^38^, ^39^ ^]^ The samples deposited in the air show a reduced intensity of the diffraction peaks, which can be attributed to the lower thickness of the sample and probably to a lower crystallinity, which could have an ulterior influence on the AVT values as a higher crystallinity can cause dispersion of light at the grain border, thus reducing the transmittance, while a partial structural disorder may promote higher transmittance instead.^[^ ^40^ ^]^ A negligible amount of hexagonal delta‐phase is found in the 1 m sample fabricated in the glovebox, identifiable with the peak at 11.80°.^[^ ^41^ ^]^

**Figure 3:**
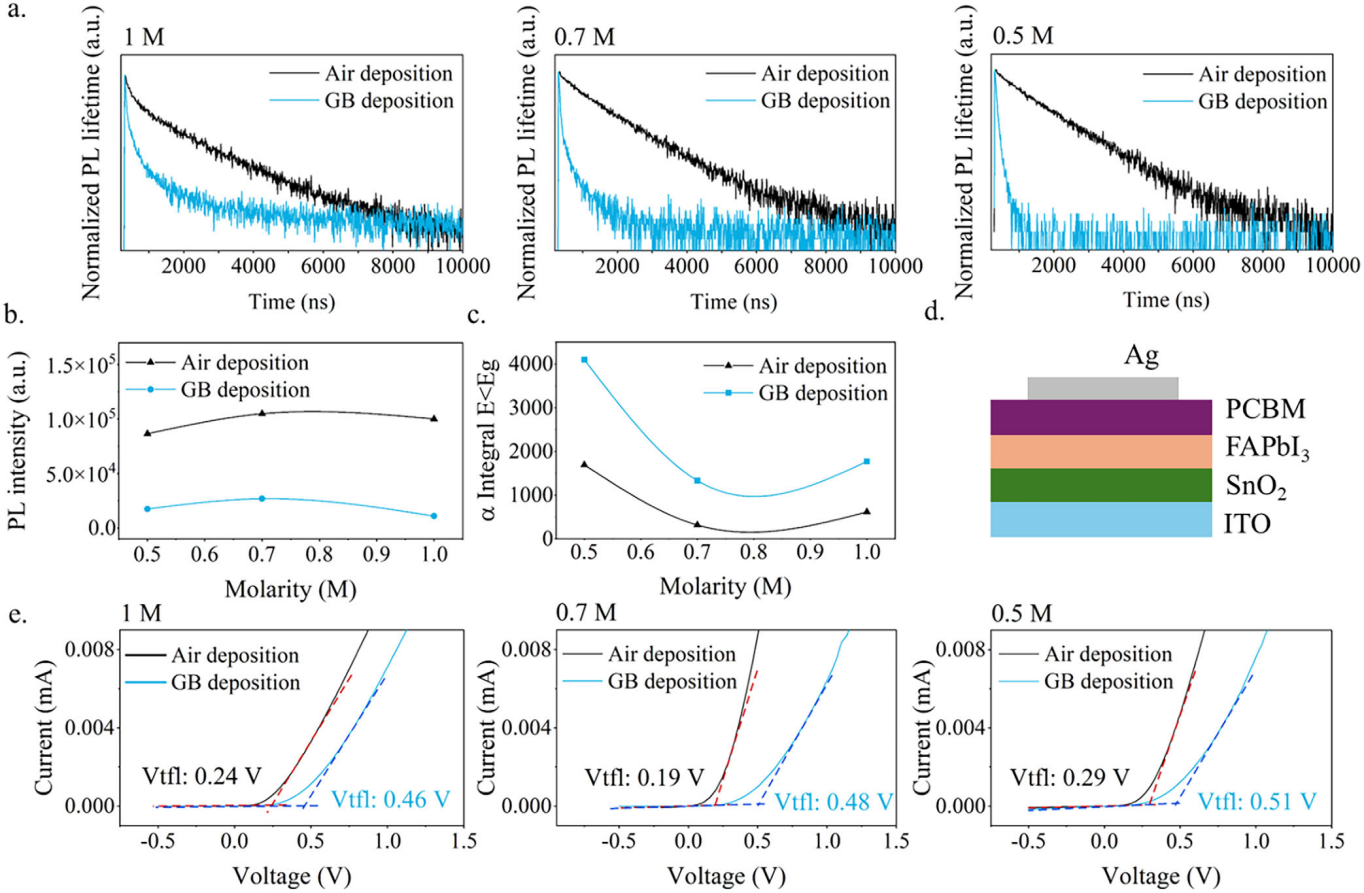
a) Photoluminescence lifetime of FAPbI_3_ perovskite films with different concentrations on glass substrate deposited in glovebox and in ambient air b) Photoluminescence intensity values versus the molarity of the perovskite c) α Integral E<Eg versus the molarity of the perovskite d) Sketch of the only‐electron device e) *J*–*V* dark curves of FAPbI_3_ with different concentrations deposited in glovebox and in ambient air acquired on electron only devices.

To further investigate the optical characteristics of the FAPbI_3_ samples, complementary techniques were used to assess the effect of the deposition in ambient air on defect formation. First, Photoluminescence (PL) and Time‐Resolved PL (TRPL) spectra were acquired. In **Figure** **3**a–c the PL lifetimes of all the films are shown and, surprisingly, all the samples prepared in ambient air condition are characterized by extremely longer PL lifetimes, which are reported in Table S2 (Supporting Information), compared to the samples deposited in the glovebox. The enhancement of PL lifetime, as well as PL intensity reported in Figure 3d and Figure S8 (Supporting Information), is connected to a smaller number of defects in the film, which leads to reduced recombination centers and a more efficient radiative recombination,^[^ ^42^, ^43^ ^]^ coherently with the beneficial effect of moisture during the deposition phase which not only promotes the growth of larger grains but also facilitates a slower crystallization with fewer defects, likely due to the enhanced mobility of the organic cation.^[^ ^30^, ^44^ ^]^

To better understand the properties of the samples, spectroscopic ellipsometry measurements were conducted to determine the refractive indexes and calculate the absorption coefficients (Figure S9, Supporting Information). The samples fabricated in ambient air show fewer defects in the gap compared to the glovebox samples (Figure 3c) since the absorption coefficients are drastically lower at energies below the gap.

To quantitively investigate the defect concentrations among the two different deposition environments, space charge‐limited current (SCLC) measurements were performed based on an electron‐only device, whose structure is shown in Figure 3d. The trap‐filled limit voltages (Vtfl) determined from the dark *J*–*V* curves of the devices shown in Figure 3e are found to be lower in the air‐fabricated devices for each perovskite concentration. The trap densities (n_t_) are calculated accordingly^[^ ^45^, ^46^ ^]^ and reported in the Supporting Information. Notably, all the n_t_ values for the devices fabricated in the air are lower, consistent with the PL, TRPL, and absorption coefficient measurements, but the difference is exacerbated for the 0.7 m concentration, for which the n_t_ are 5.93E^+16^ cm^−3^ for the air‐fabricated and 1.10E^+17^ cm^−3^ for the glovebox‐fabricated electron only devices (Table S3, Supporting Information).

**Figure 4:**
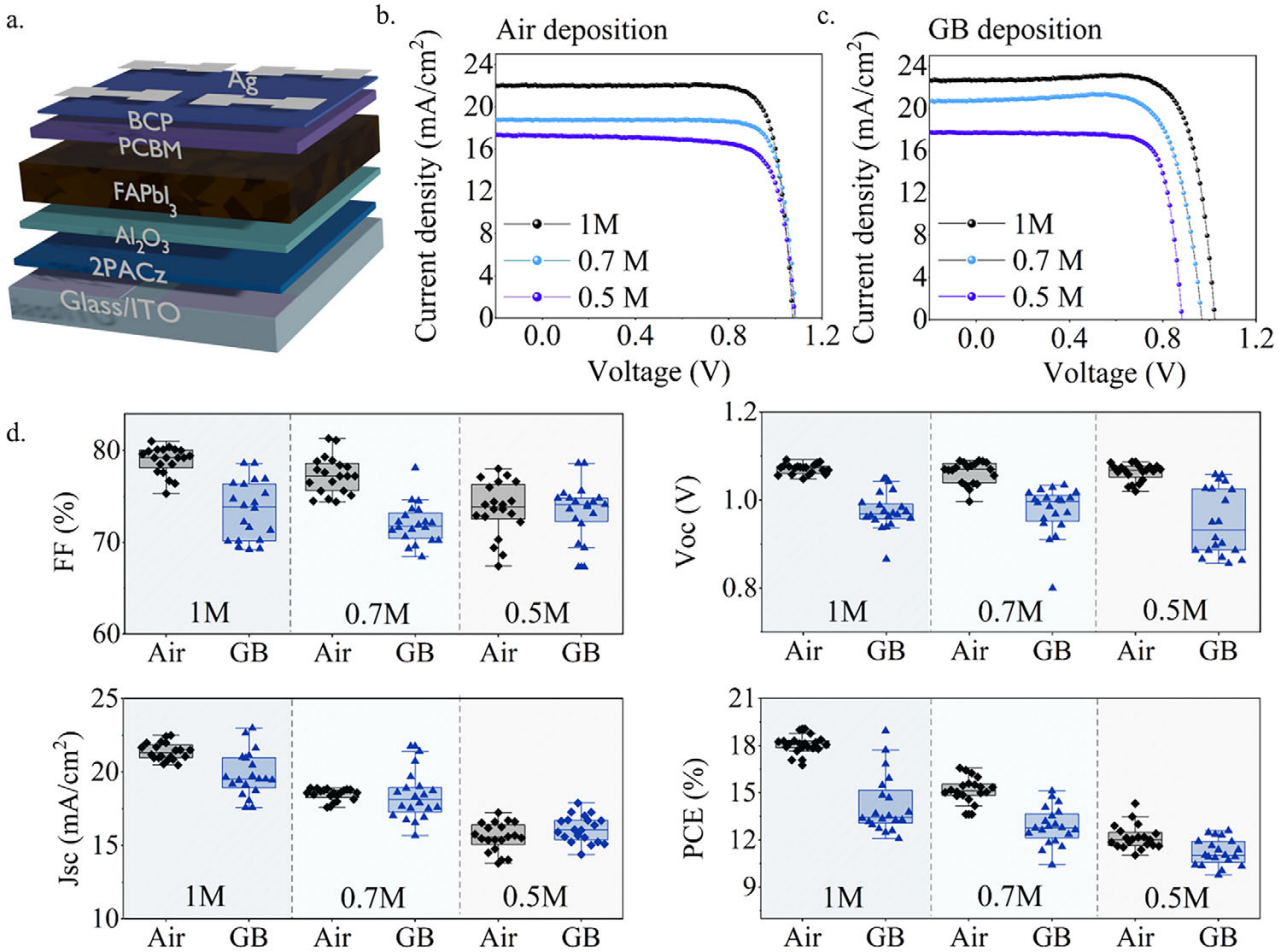
a) Sketch of the device architecture b) *J*–*V* curves of FAPbI_3_ devices fabricated in air c) *J*–*V* curves of FAPbI_3_ devices fabricated in GB d) Statistical photovoltaic parameters of FAPbI_3_ devices with different concentrations deposited in air and in the glovebox.

To finally address the beneficial effects of ambient air deposition on the actual PSCs, *p*‐*i*‐*n* devices were fabricated in inverted architecture, as sketched in **Figure** **4**a first using Ag opaque back electrodes. The devices with the active layer fabricated in the air show higher performances compared with the glovebox‐fabricated devices (Figure 4b,c). Moreover, the *J*–*V* curves of the glovebox‐fabricated devices show a current maximum above the Jsc or “bump” close to the Voc.

These bumps are associated with excessive ionic mobility caused by I^−^ vacancies^[^ ^47^, ^48^ ^]^ and charge accumulation phenomena between the perovskite layer and the charge transporting layers upon bias,^[^ ^49^, ^50^ ^]^ meaning that charge extraction is facilitated in the devices fabricated in the air with respect to the devices fabricated into the glovebox. Moreover, the glovebox‐fabricated devices exhibit higher hysteresis (Figure S10, Supporting Information), further supporting the observation of greater defect density and excessive ionic mobility.^[^ ^51^ ^]^ The incident photon‐to‐current conversion efficiency (IPCE) spectra are reported in Figure S11 (Supporting Information), along with the integrated current which are both coherent with the measured ones in the *J*–*V* curves. The IPCE spectra for all the concentrations of the devices fabricated in the air show a particular shape with higher quantum efficiency at lower wavelengths, while, near the bandgap at higher wavelengths, the efficiency is lower compared to the glovebox devices. This can be explained considering that the regime in the IPCE spectrum with λ > 500 nm is demonstrated to be strongly influenced by the thickness of the active layer, which is higher for the FAPbI_3_ deposited in the glovebox, causing the optical interferences.^[^ ^52^, ^53^ ^]^ Instead, in the Beer‐Lambert regime with λ < 500 nm, the carriers are photogenerated near the surface, meaning that the air‐fabricated devices show fewer recombination losses compared to the glovebox devices_,_^[^ ^54^ ^]^ confirming the previous observations. The statistical photovoltaic parameters of all the concentrations are shown in Figure 4d and Table S4 (Supporting Information), and the superior performances of the air devices are highlighted. Specifically, the Voc values, which are maintained high and constant regardless of the perovskite concentration, are crucial for the enhancement of PCE. The improvement in Voc is coherent with the previous discussion on the lower defect concentration found in the air‐fabricated FAPbI_3_, and the distribution of values is visibly smaller, highlighting better reproducibility and a superior uniformity of the samples. Instead, the differences found in the fill factor (FF) and short circuit current density (Jsc) values are smaller, especially the Jsc values which are similar and, as expected, are decreasing with the perovskite concentration and thicknesses.

To evaluate the stability of the device maximum power point (MPP) measurements were performed following the ISOS‐L‐1 protocol^[^ ^56^ ^]^ in ambient air (Figure S12a, Supporting Information) with light‐dark cycles of 8–16h. The devices fabricated in the air are found to be more stable, showing a slower decrease in efficiency over time, especially the 1 m concentration which is extremely stable over time maintaining 100% of the initial efficiency after 4 cycles. Instead, all GB‐fabricated samples go below 80% of the efficiency after 1 cycle. To confirm the superior stability of the air‐fabricated FAPbI_3_ the shelf‐life stability was monitored following the ISOS‐D‐1 protocol.^[^ ^56^ ^]^ While the devices with the GB deposition suffer a drastic drop of 50% in PCE after 24 h, the air‐fabricated devices retain 80% of the initial efficiency after 15 days. The 1 m sample fabricated in air is confirmed to be the most stable (Figure S12b, Supporting Information).

**Figure 5:**
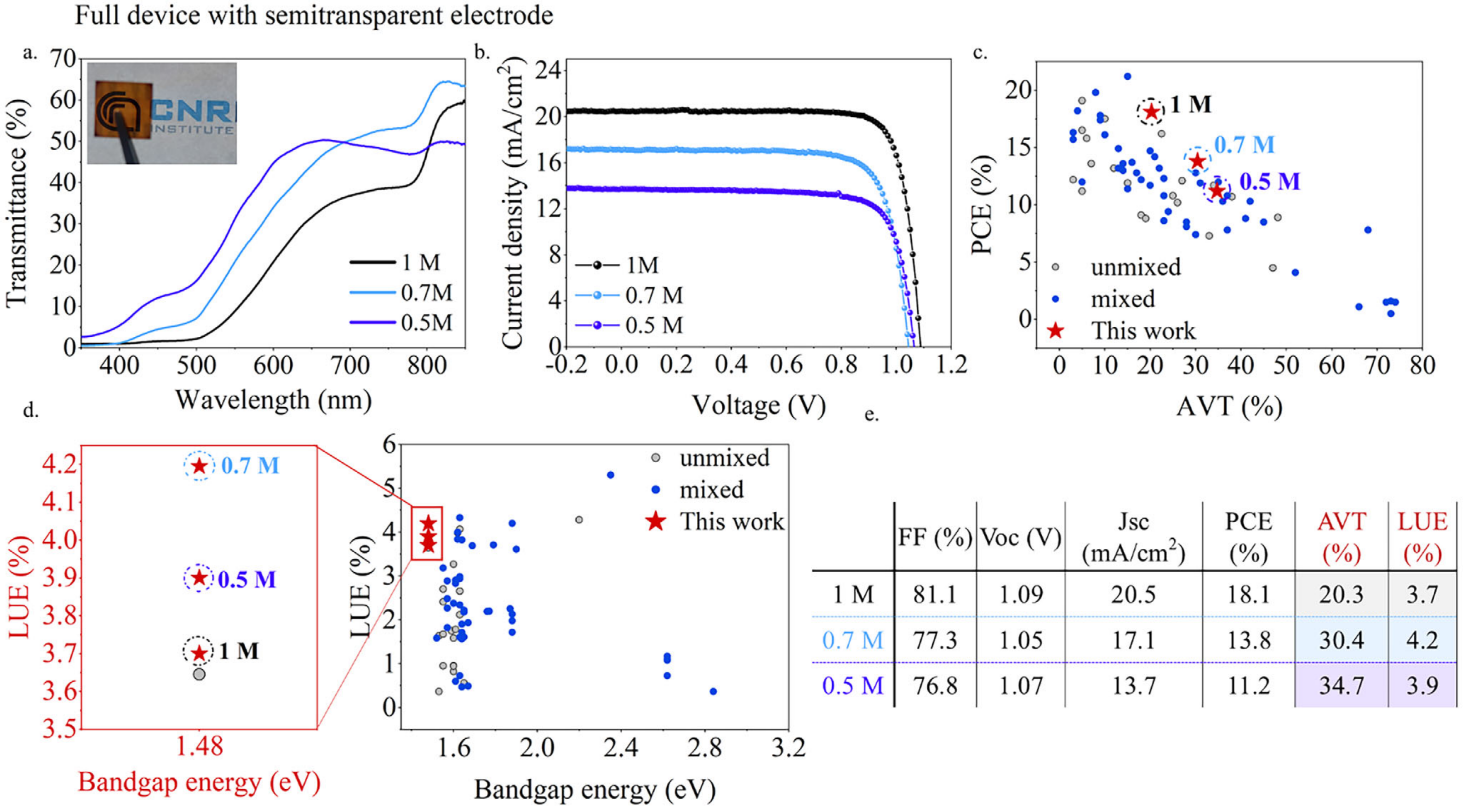
a) Transmittance spectra of the full devices with semi‐transparent Au electrode b) *J*–*V* curves of FAPbI_3_ devices deposited in the air with semi‐transparent Au electrode c) PCE (%) as a function of AVT (%) for semi‐transparent perovskite solar cells with mixed and unmixed composition in literature^[^ ^20^, ^55^ ^]^ d) LUE (%) as a function of bandgap energy (eV) for semi‐transparent perovskite solar cells with mixed and unmixed composition in literature^[^ ^55^ ^]^ with focus on 1.48 eV^[^ ^20^ ^]^ e) Photovoltaic parameters of best devices with AVT (%) and LUE values.

Finally, fully semi‐transparent devices are fabricated with the air‐deposited FAPbI_3_ evaporating 10 nm thin Au as an electrode. The transmittance spectra of the complete device stack and *J*–*V* curves are shown in **Figure** **5**a,b and clearly show the same trend as the opaque devices. In Figure 5c–e the performances of the best devices are shown together with AVT and LUE value and confronted with literature reports. As shown in Figure 5c, high PCE values are achieved for the corresponding AVT values outperforming the present literature data and, while the LUE values shown in Figure 5d,e are high for all the concentrations, the 0.7 m shows the record high value of LUE of 4.2%, which is the highest reported value for this bandgap.

## 3 Discussion

In summary, an unprecedented LUE value for FAPbI_3_ has been reached by depositing the perovskite in ambient air, which allows a great enhancement of the optical properties and, consequently, of the AVT of the perovskite without compromising the device's efficiency and stability. The presence of moisture during the perovskite deposition is found to be crucial to enlarging the grain size, effectively increasing the transparency of the film, and reducing the perovskite's defects, overall obtaining an enhanced perovskite optical and optoelectronic quality. The devices with the air‐fabricated perovskite possess superior PCEs and stability. This straightforward and general approach allows us to combine higher AVT with enhanced performance, maximizing the LUE value and going toward the market requirements for semi‐transparent photovoltaics.

## 4 Experimental Section

### Chemicals

Lead (II) iodide (PbI_2_, ultradry 99.999% metals basis) was purchased from Alfa Aesar (Kandel, Germany); Formamidinium iodide (FAI, >99.99%); Methylammonium chloride (MACl) was from Sigma–Aldrich; Chlorobenzene anhydrous, 99.8% (CB); 2‐propanol (IPA); bathocuproine, 96% (BCP), Aluminium oxide (nanoparticles 20 wt.% in water), 2PACz [2‐(9H‐Carbazol‐9‐yl)ethyl]phosphonic Acid, >98.0% was purchased from TCI; [6,6]‐phenyl C61 butyric acid methyl ester (PCBM) was purchased from Nano‐c.

### Perovskite Solutions Preparation

The FAPbI_3_ 1 m solution was prepared by dissolving 461 mg of PbI_2_ (1 mmol), 172 mg of FAI (1 mmol), 13.5 mg of MACl (0.2 mmol) in 1 mL of the solvent mixture DMF:NMP (9:1). The 0.7 and 0.5 m solution were diluted from the 1 m.

### Spin Coater Device Fabrication

The glass ITO patterned substrates (15×15 mm^2^) were sequentially cleaned by ultrasonication in deionized water, acetone, and isopropanol for 10 min each. The ITO substrates were dried with nitrogen and UV‐Ozone treatment was before the hole transport layer (HTL) deposition. 2PACz (0.5 mg mL^−1^ in ethanol) solution was spin‐coated in the glovebox on ITO substrates at 3000 rpm for 30 s and annealed at 100 °C for 10 min. After cooling them down, the substrates were washed with a solution of 0.2 wt.% of Al_2_O_3_ nanoparticles in ethanol to remove the unbound molecules and then annealed again at 100 °C for 5 min. The perovskite solutions were spin‐coated in air and a glovebox at 5000 rpm x 19 s and 400 µL of ethyl acetate was dropped on the spinning substrate at 11th second. The substrates were annealed for 10 min at 160 °C. 50 nm of the ETL were deposited in the glovebox through the spin coating of the PCBM solution (25 mg mL^−1^ in chlorobenzene) at 1000 rpm for 60 s. A thin layer of BCP (0.5 mg mL^−1^ in isopropanol) was then deposited by spin coating at 6000 rpm for 20 s. Finally, 80 nm of Ag electrodes (10 nm of Au electrode for the semi‐transparent devices) were thermally evaporated in a high vacuum with an active area of 0.04 cm^2^. The devices were characterized using a Keithley 2400 Source Measure Unit and Air Mass 1.5 Global (AM1.5G) solar simulator (Newport 91160A) exposed to irradiation intensity of 100 (mW cm^−2^). The Current–voltage curves were acquired in the range from 1.2 to −0.2 V.

For the electron‐only devices, SnO_2_ nanoparticles 15 wt.% in water were diluted to 2.5 wt.% in H_2_O:IPA 80:20 and spin‐coated at 2000 rpm for 30 s. The substrates were then annealed at 120 °C for 20 min.

### Characterization

The SEM, Transmittance, and Photoluminescence measurements were conducted as described in a previous report.^[^ ^24^ ^]^

### Characterization—Spectroscopic Ellipsometry Analyses were used to Calculate the Absorption Coefficient

Spectroscopic Ellipsometry (SE) data were collected using a J. A. Woollam VASE instrument. Measurements were performed in a vertical configuration, which was better suited for transparent samples to measure on the same point ellipsometric and transmittance data. Optical spectra were recorded from 190 to 2500 nm (0.5–6.3 eV) at 55°, 60°, 65° and 70° below and above the Brewster angle. The layer was modeled by using multiple CPPB (Critical Point Parabolic Band) oscillators. Special care has been used to evaluate the optical constants for the glass substrate, taking into account backside reflection and unpolarised light.

### Characterization—X‐Ray Diffraction (XRD)

Patterns were obtained utilizing a SmartLab (Rigaku) diffractometer fitted with a 9 kW rotating anode Cu X‐ray source, running at 45 kV and 200 mA, in conjunction with a HyPix‐3000 detector. The recording step size for the patterns was set at 0.02°, with an acquisition speed of 1° min^−1^. The analyses were performed in N_2_ ambient using an Anton Paar DHS 1100 substrate holder equipped with a PEEK dome.

### Atomic Force Microscopy

The surface morphology was examined by using tapping‐mode atomic force microscopy (Veeco, Multimode IIID Microscope). The perovskite was deposited onto ITO/2PACz/Al_2_O_3_ substrates.

## Conflict of Interest

The authors declare no conflict of interest.

## Supporting information



Supporting Information

## Data Availability

The data that support the findings of this study are available from the corresponding author upon reasonable request.
