# Supplementary material for: Ambient Air Deposition Allows Reaching Record Light Use Efficiency in FAPbI3 Perovskite Solar Cells
Source: Adv Sci (Weinh). 2025 May 8;12(25):2501533. doi: 10.1002/advs.202501533 (PMC12225017; doi:10.1002/advs.202501533)
Supplement: Supplementary file 1 — Supporting Information [file ADVS-12-2501533-s001.docx]

**Ambient Air Deposition Allows Reaching Record Light Use Efficiency in FAPbI_3_ Perovskite Solar Cells**

Nadir Vanni^1,2^, Mario Calora^1,2^, Lucia Mercurio^1,2^, Antonella Giuri^2^, Annapaola Caricato^1^, Veronica Chierchia^3^, Claudio Carati^3^, Riccardo Po'^3^, Paolo Biagini^3^, Salvatore Valastro^4^, Emanuele Smecca^4^, Giovanni Mannino^4^, Alessandra Alberti^4^ and Aurora Rizzo ^2^.

^1^Dipartimento di Matematica e Fisica “E. De Giorgi”, Università del Salento, Campus Ecotekne, via Arnesano, 73100 - Lecce (Italy)

^2^CNR NANOTEC – Istituto di Nanotecnologia, c/o Campus Ecotekne, Via Monteroni, 73100 - Lecce (Italy)

^3^Renewable, New Energy and Material Science Research Center, Istituto Guido Donegani, Eni S.p.A., via Fauser 4, I-28100 - Novara (Italy)

^4^CNR-IMM, Zona Industriale Strada VIII, 5 - 95121 Catania, Italy

**Supporting Information**

Table S1. Thickness values of FAPbI_3_ samples deposited in glovebox and in air with calculated ΔThickness and ΔAVT

**
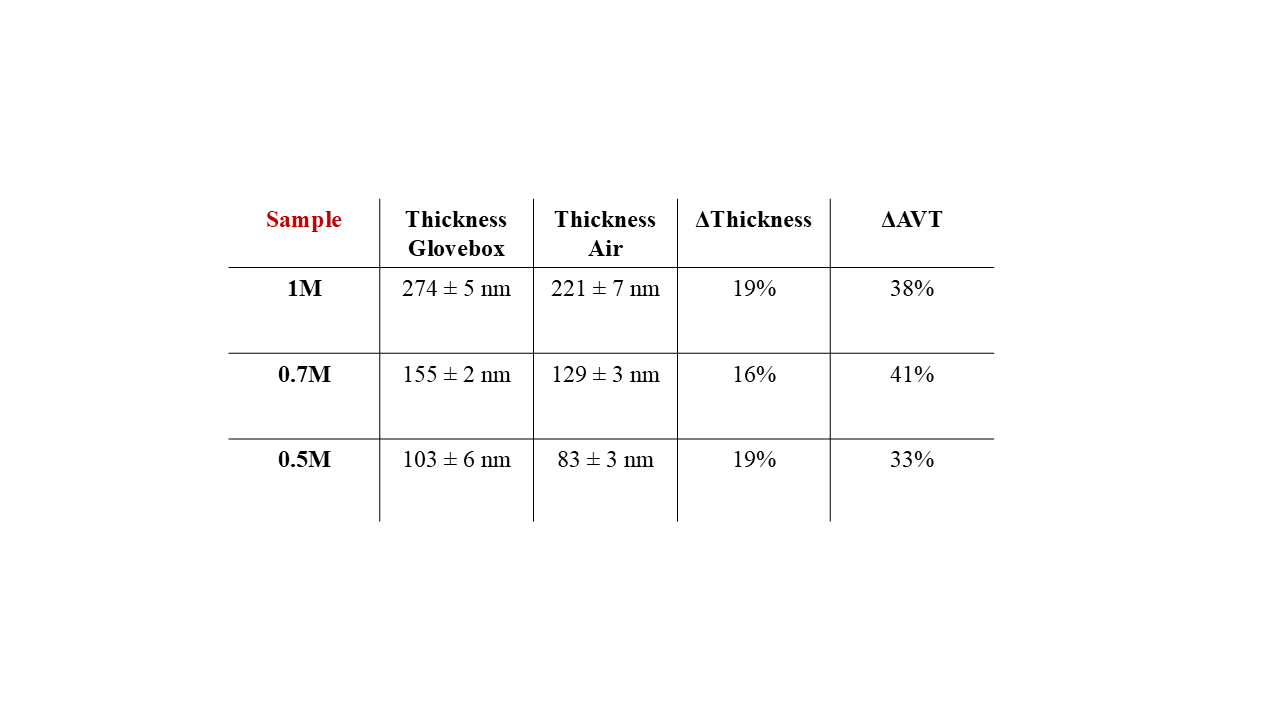
**

**
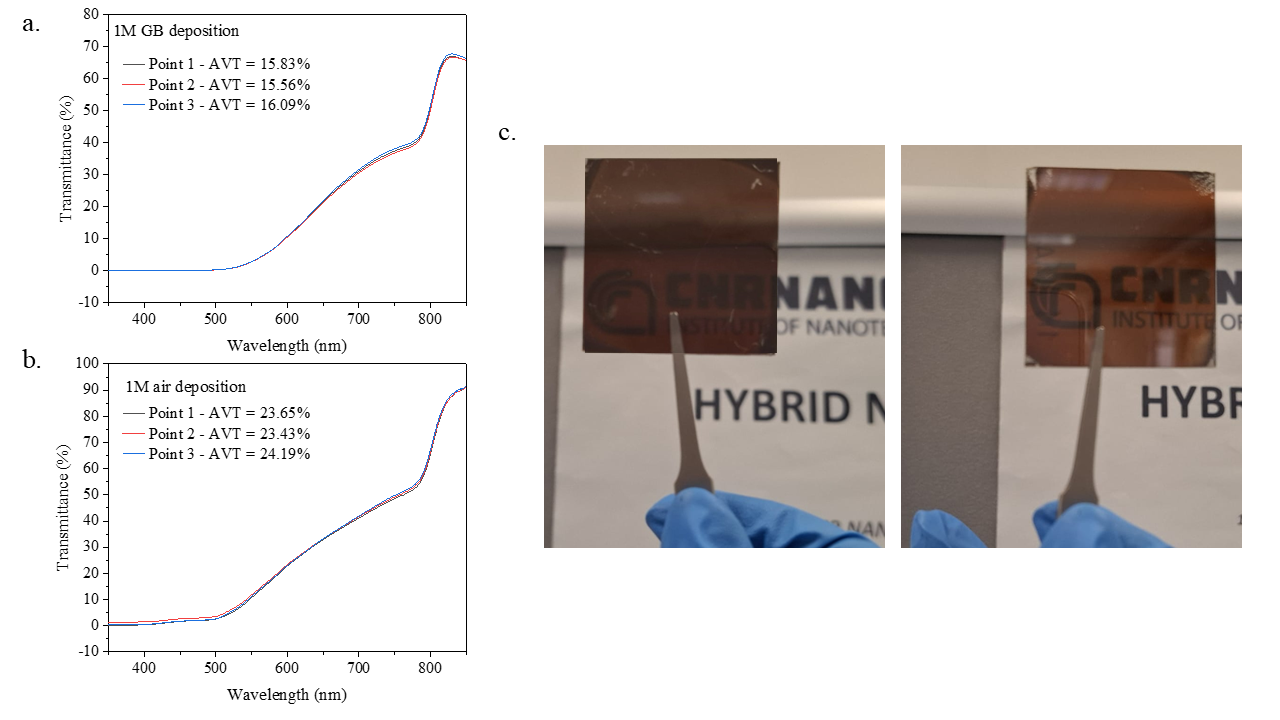
**

Figure S1. Transmittance spectra of FAPbI_3_ 1 M perovskite films on glass substrate deposited in a) glovebox and in b) ambient air taken in three different spots. c) Photos of the samples


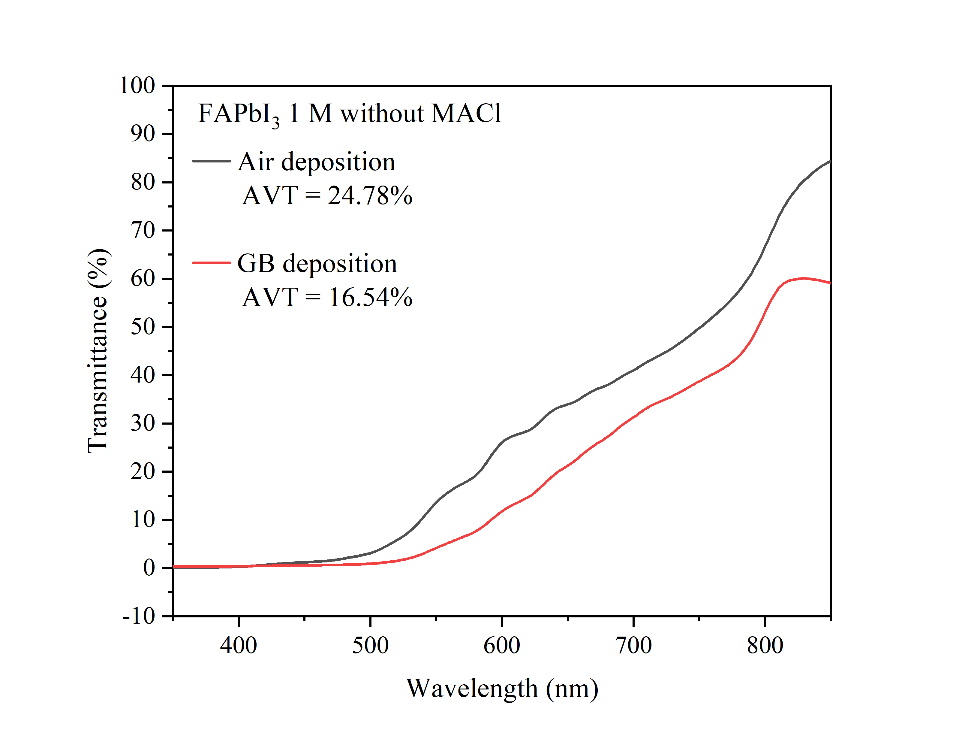


Figure S2. Transmittance spectra with calculated AVT(%) of 1 M sample without MACl deposited in ambient air and in glovebox


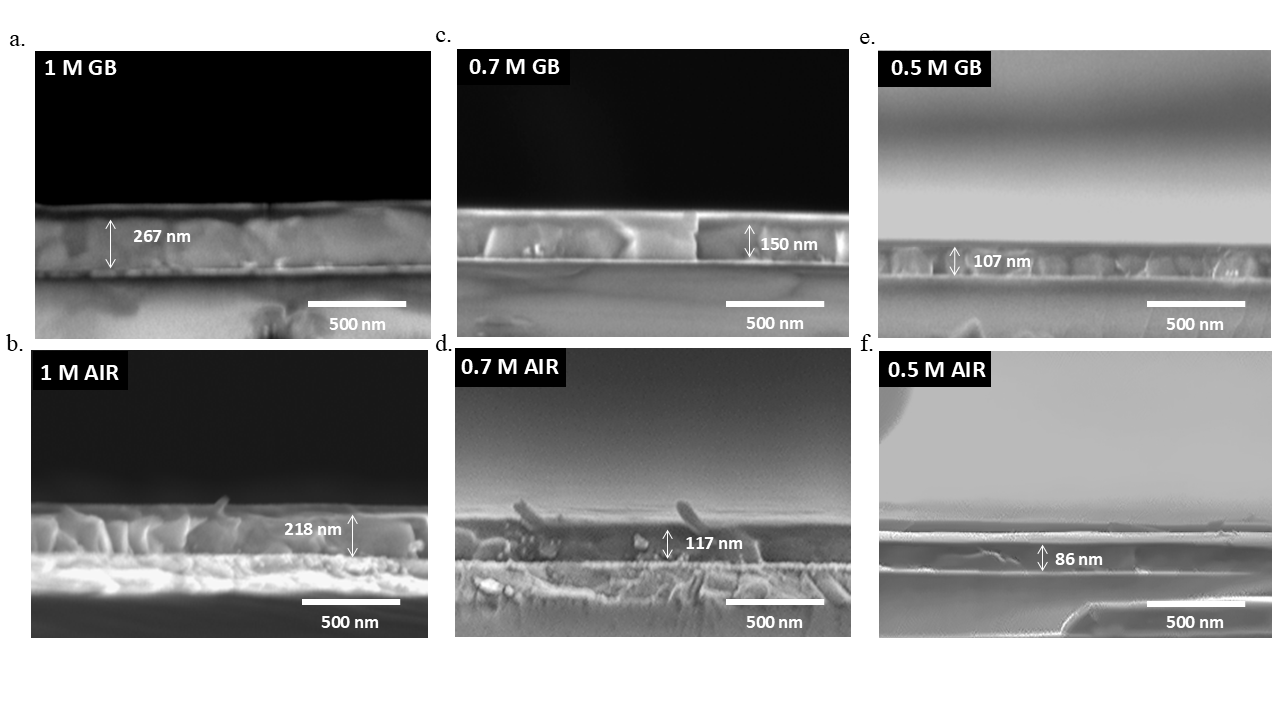


Figure S3. Cross-sectional SEM images of devices based on a) FAPbI_3_ 1 M deposited in the GB b) FAPbI_3_ 1 M deposited in the air c) FAPbI_3_ 0.7 M deposited in the GB d) FAPbI_3_ 0.7 M deposited in the air e) FAPbI_3_ 0.5 M deposited in the GB f) FAPbI_3_ 0.5 M deposited in the air

**
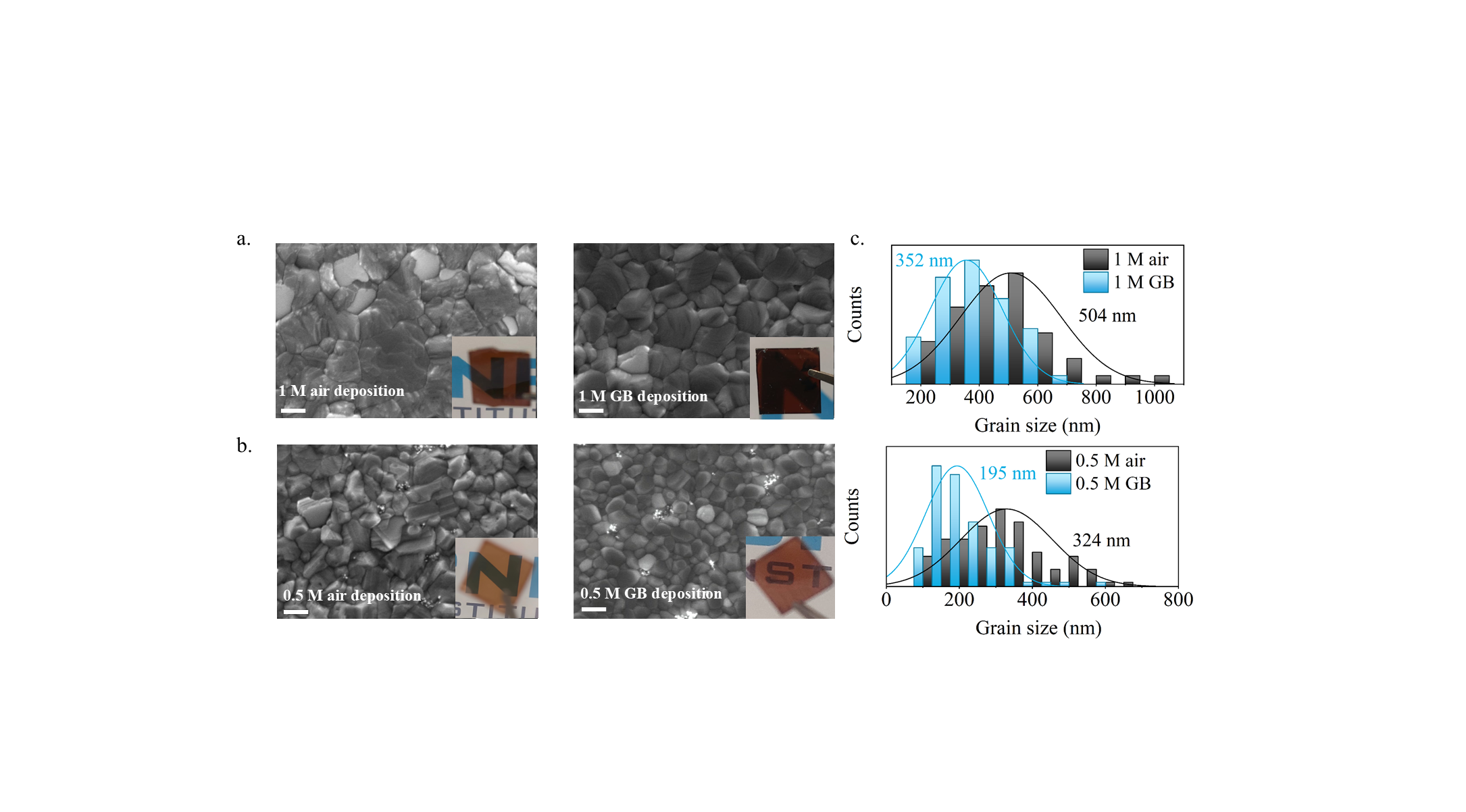
**

Figure S4. a) SEM image (scale 300 nm) of FAPbI_3_ 1 M deposited in the air and in GB b) SEM image (scale 300 nm) of FAPbI_3_ 0.5 M deposited in air and in GB c) Grain size distribution of 1M and 0.5 M FAPbI_3_ deposited in ambient air and glovebox


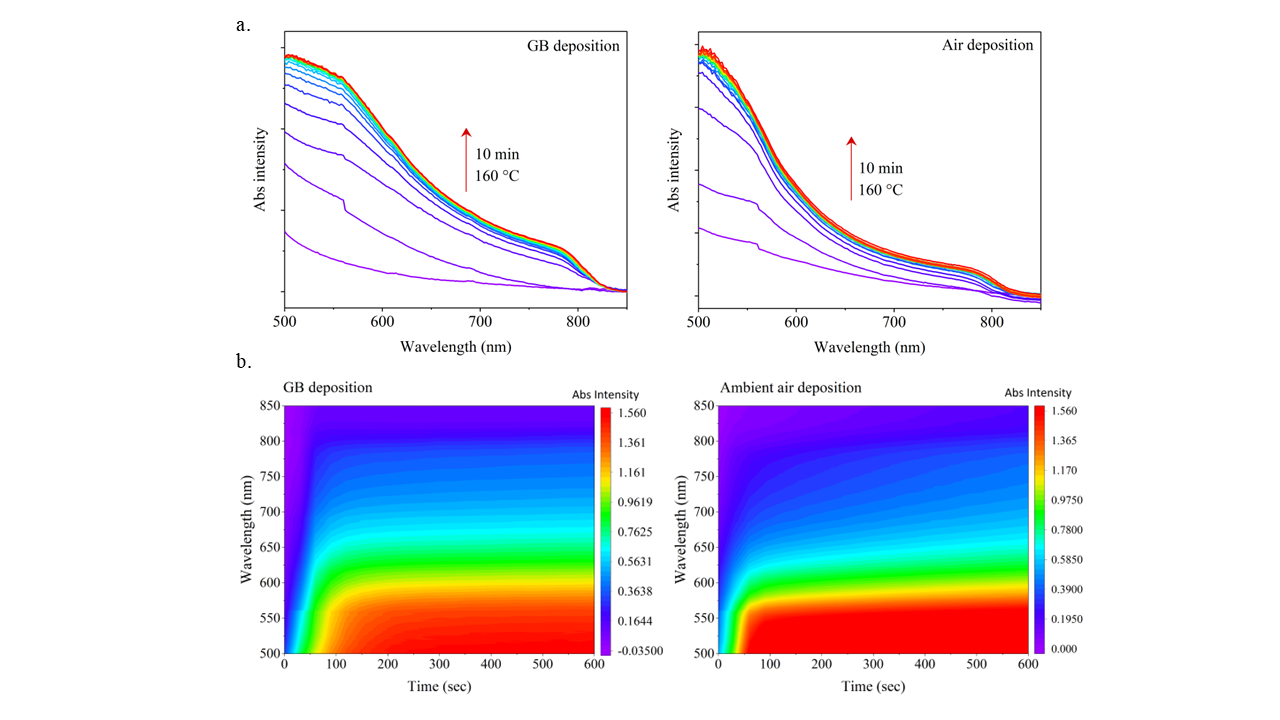


Figure S5.  a) Time-resolved absorbance spectra during annealing process of 1 M sample deposited in glovebox and in ambient air b) Corresponding colours maps


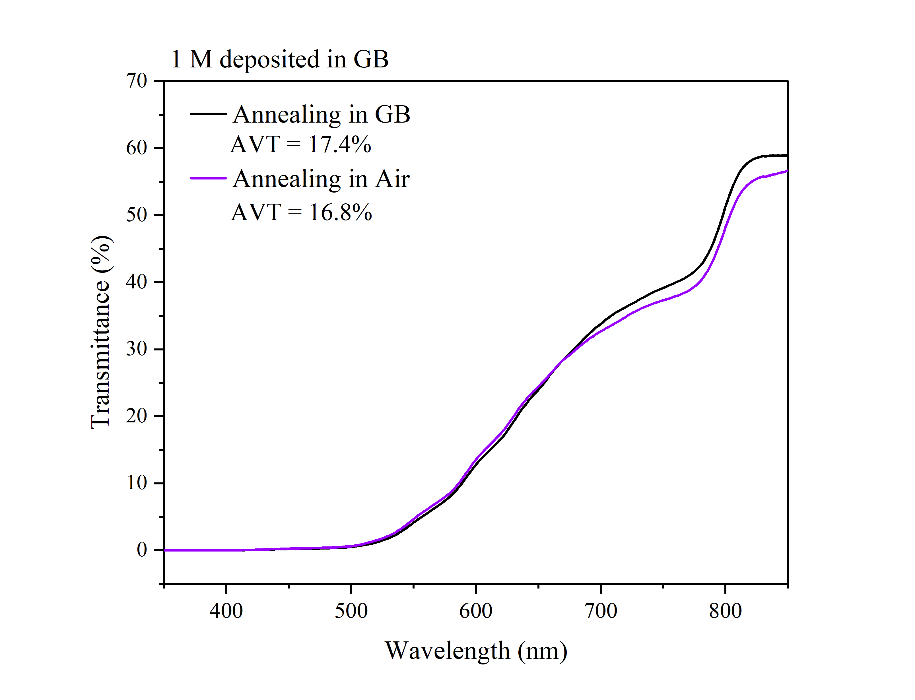


Figure S6. Transmittance spectra with calculated AVT(%) of 1 M sample deposited in glovebox with annealing in glovebox or in ambient air

**
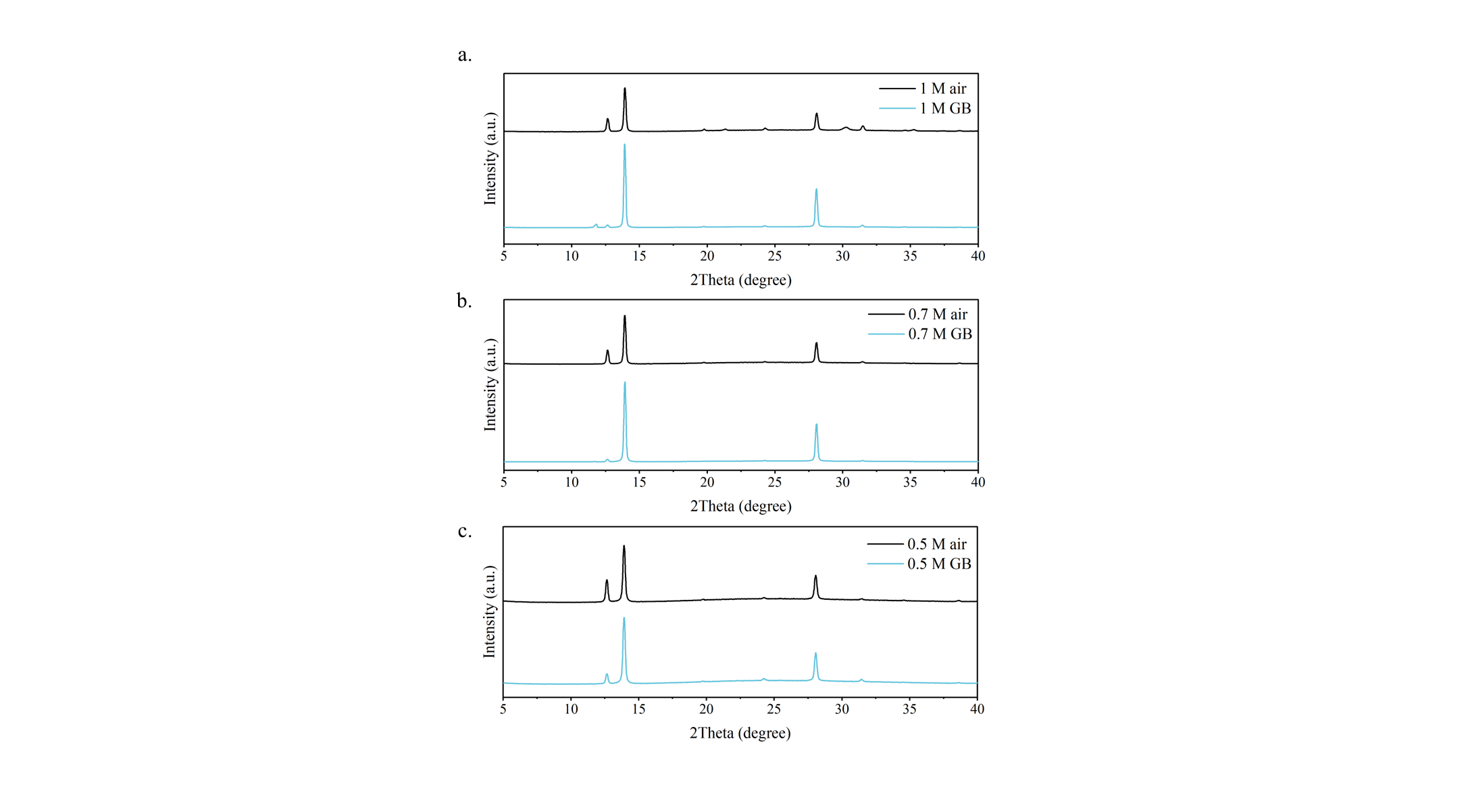
**

Figure S7. XRD patterns of samples deposited in glovebox and in air of FAPbI_3_ a)1M, b) 0.7M and c) 0.5M

Table S2. Average photoluminescence lifetimes of FAPbI_3_ samples deposited in ambient air and in glovebox.


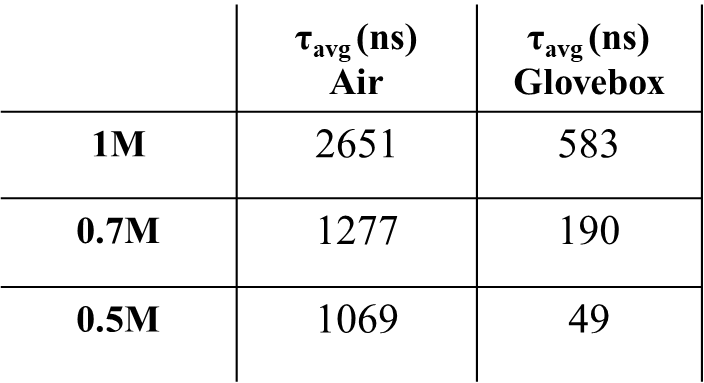


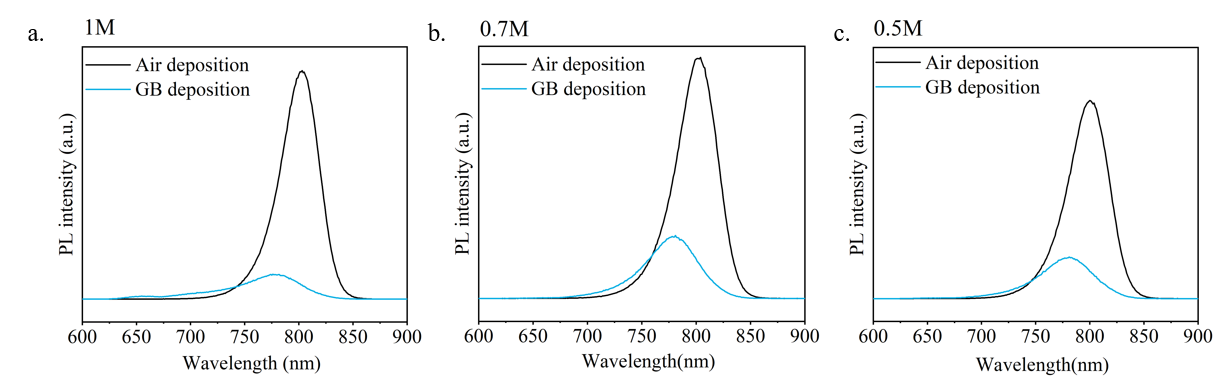


Figure S8. PL spectra of samples deposited in glovebox and in air of FAPbI_3_ a)1 M, b) 0.7 M and c) 0.5 M


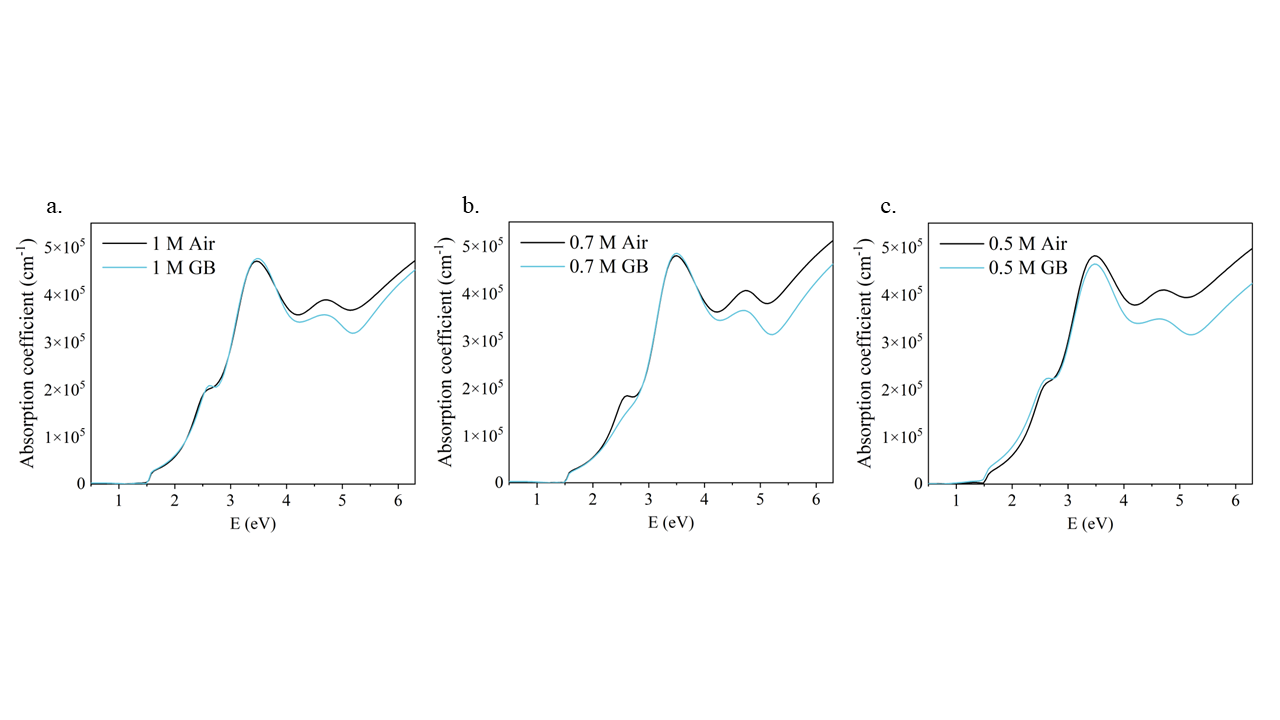


Figure S9. Absorption coefficient of samples deposited in glovebox and in air of FAPbI_3_ a)1 M, b) 0.7 M and c) 0.5 M

**Trap-state density (n_t_) calculation**

The Nt values are calculated using the trap-filled limit voltage (Vtfl) extracted from space-charge-limited current measurements following the equation reported below, where **e** is the elementary charge of the electron (1.602176634 × 10 ^−19^ coulomb), **n_t_** is the trap-state density, **L** is the perovskite film thickness, **ε** is the relative dielectric constant of perovskite, which is 47 for FAPbI_3_^1^, and **ε_0_** is the vacuum permittivity (8.85 × 10 ^−12^ F/M).

VTFL = e ∙ n_t_ ∙ L^2^ /2 ∙ ε ∙ ε_0_

Equation S1. Vtfl calculation

The n_t_ values are reported in Table S3.


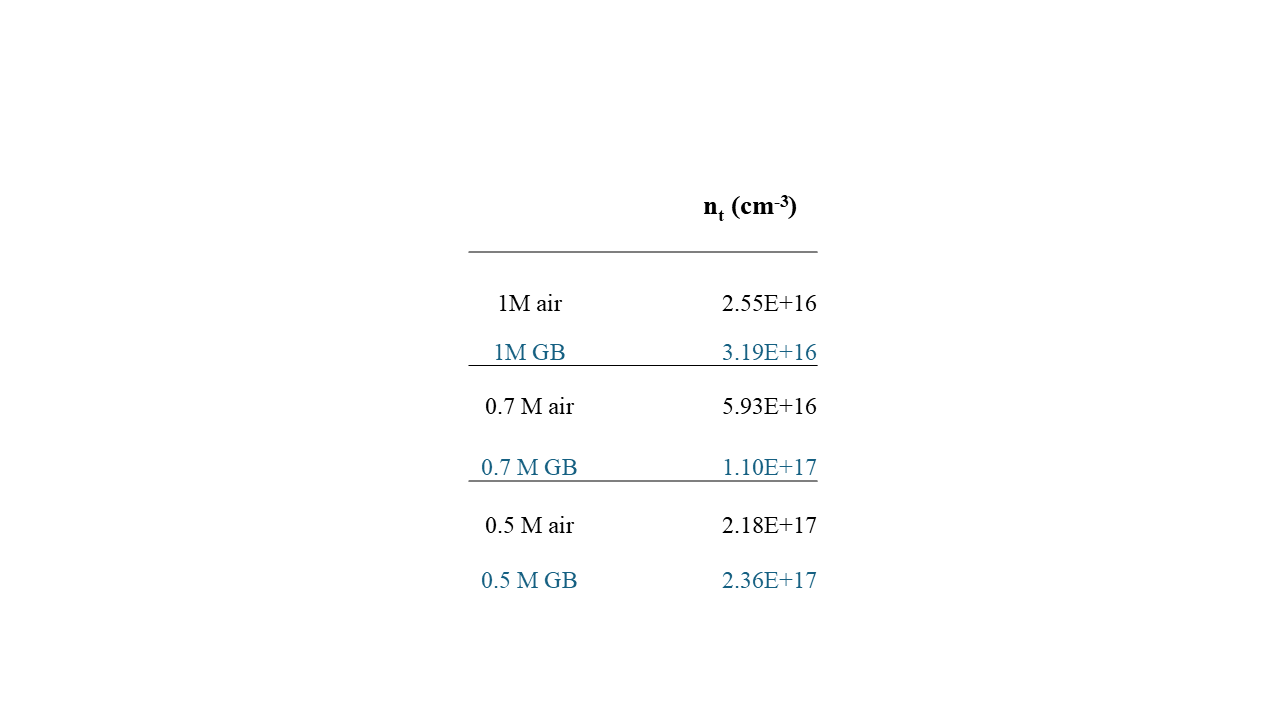
Table S3. Calculated n_t_ values of FAPbI_3_ samples deposited in glovebox and in air


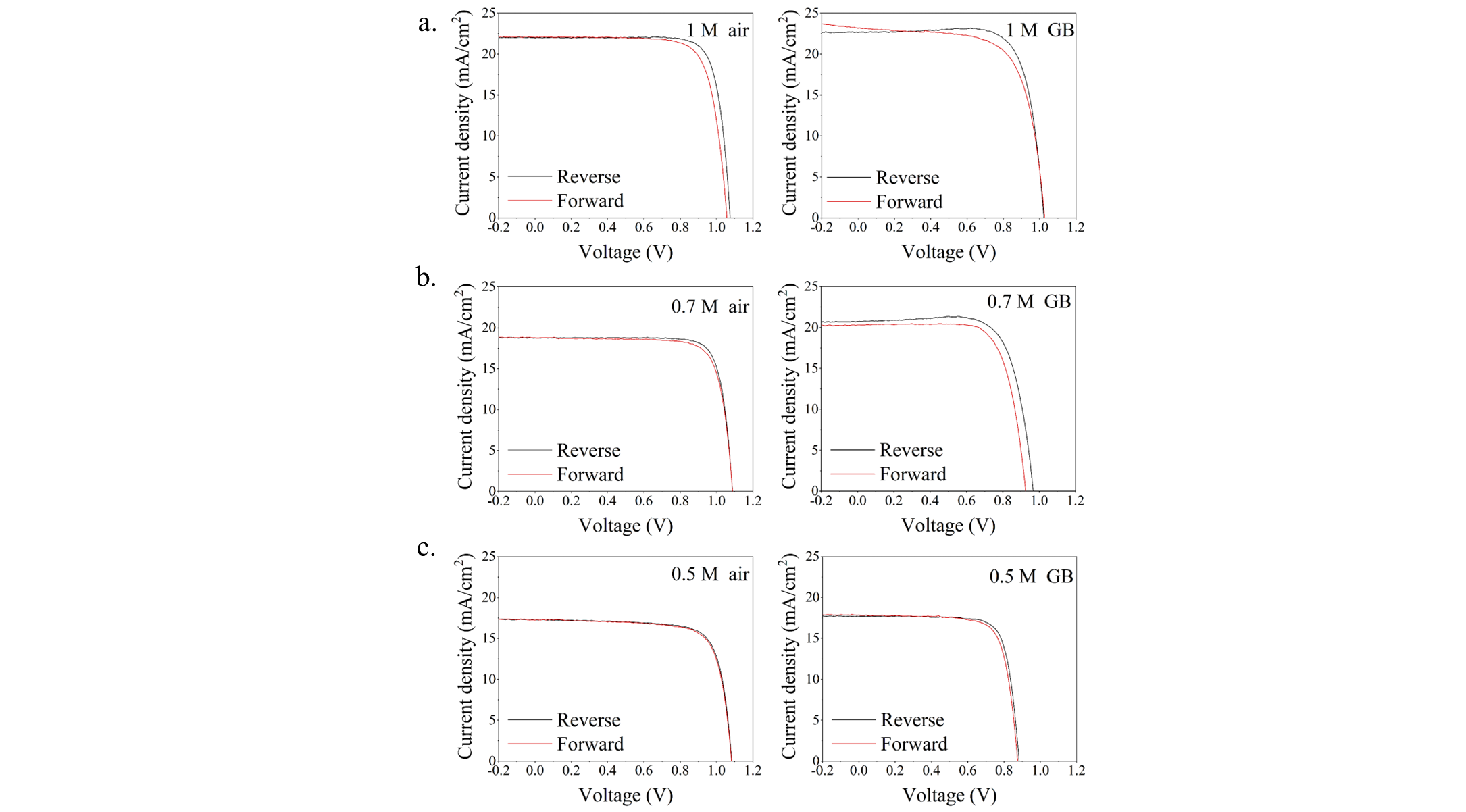


Figure S10. J-V curves in reverse and forward bias of a) 1 M FAPbI_3_ devices deposited in air and glovebox b) 0.7 M FAPbI_3_ devices deposited in air and glovebox a) 0.5 M FAPbI_3_ devices deposited in air and glovebox


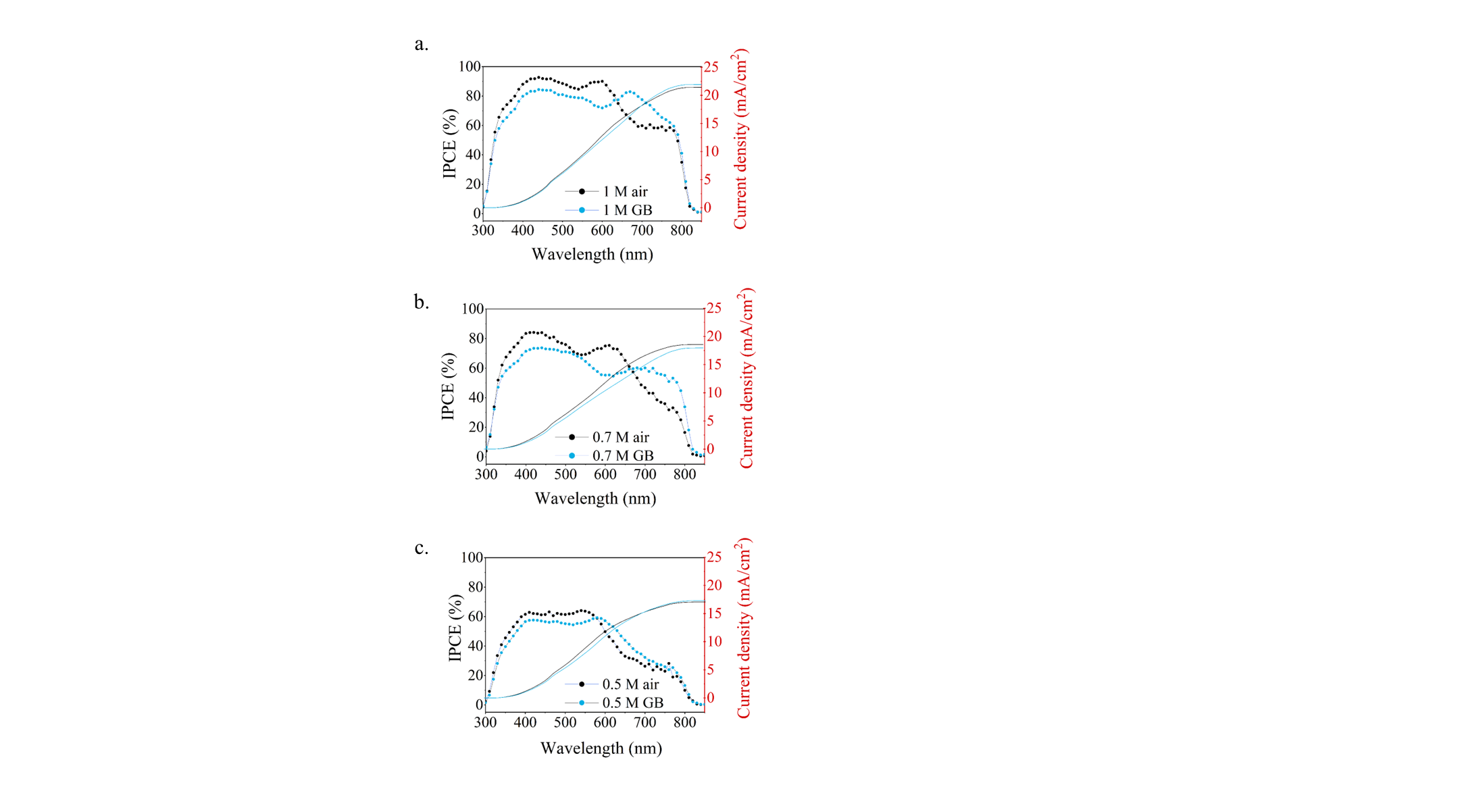


Figure S11. a) IPCE and integrated current density of 1 M devices b) IPCE and integrated current density of 0.7 M devices c) IPCE and integrated current density of 0.5 M devices


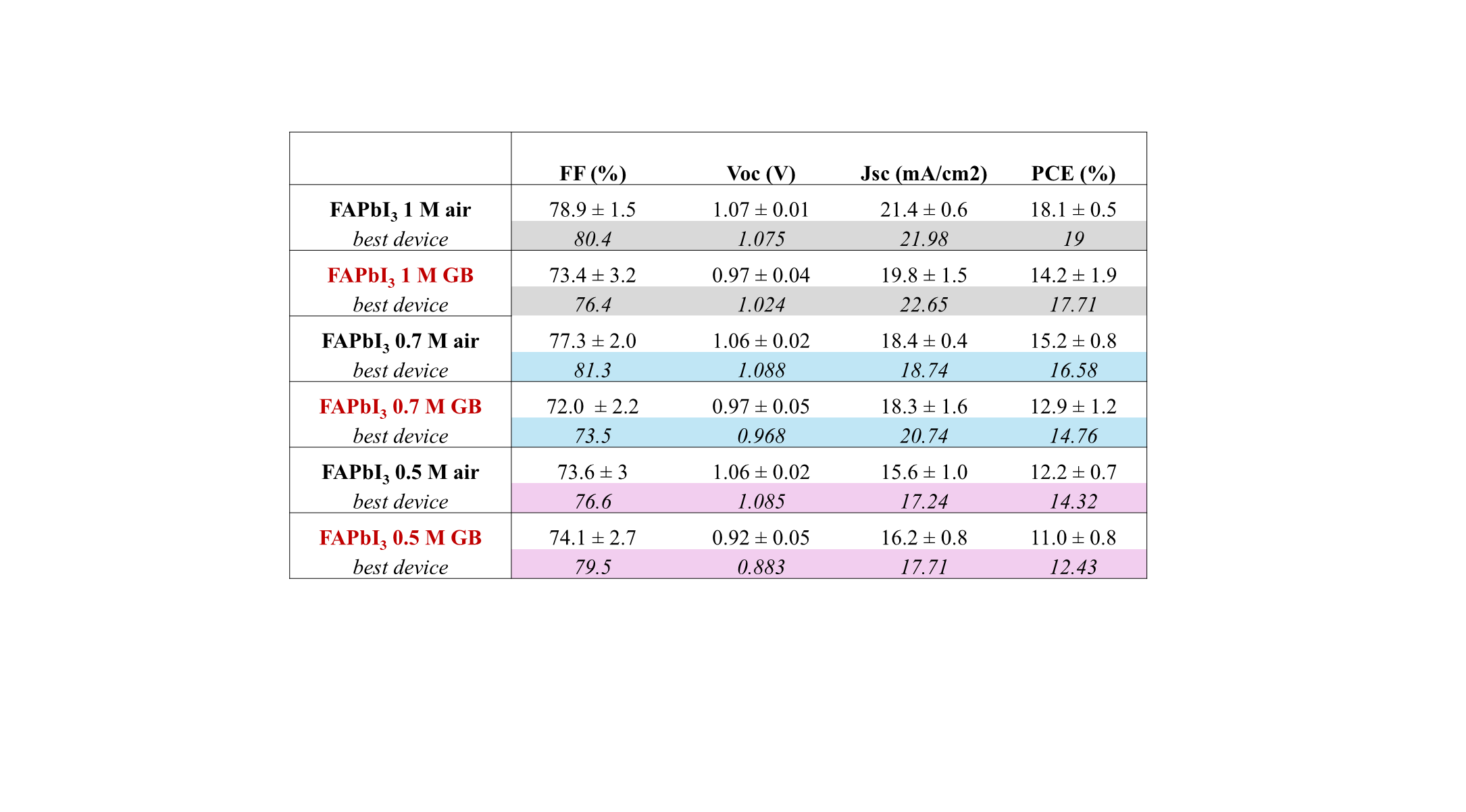
Table S4. Statistical photovoltaic parameters of FAPbI_3_ devices fabricated in air and in glovebox with different concentrations of perovskite precursors.


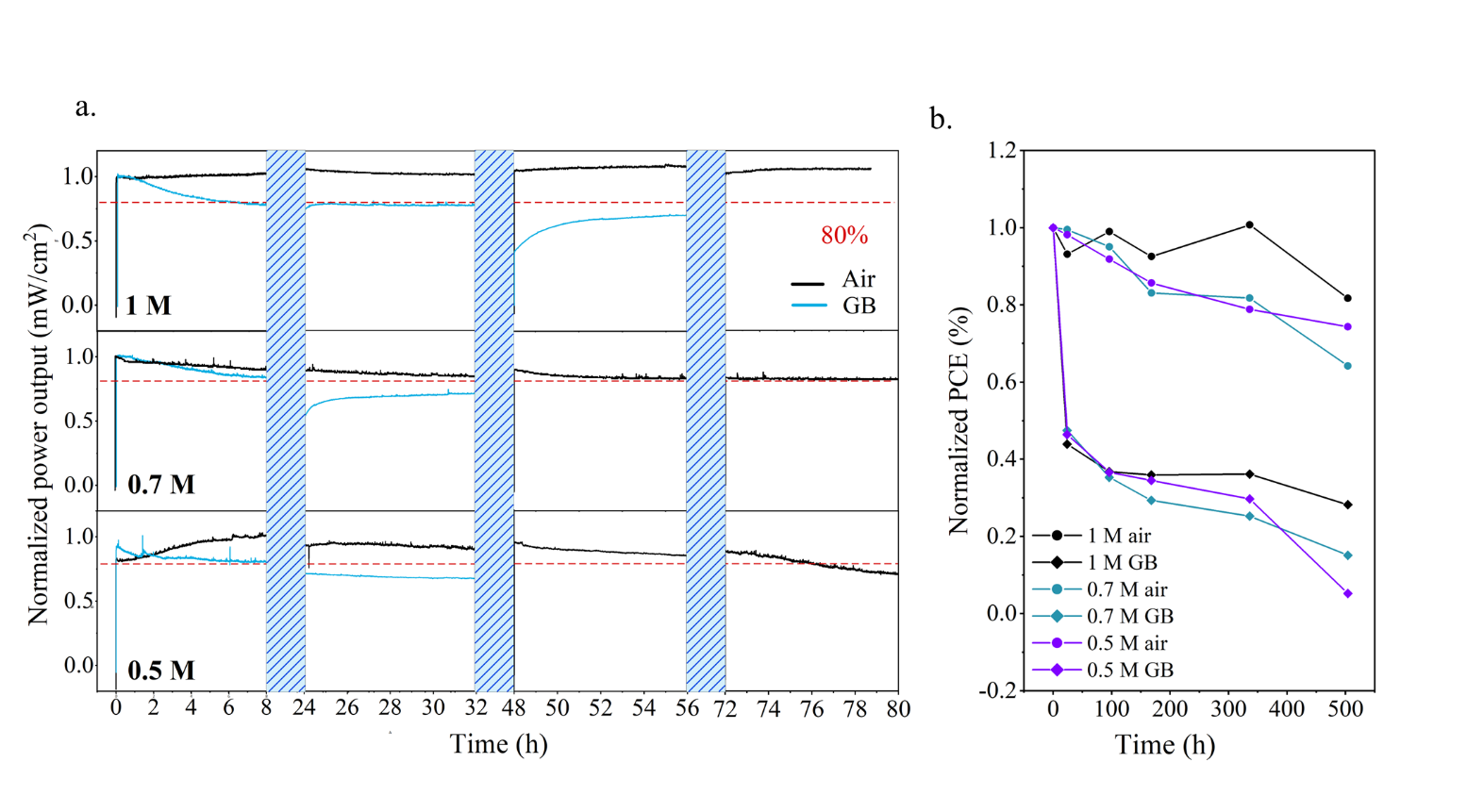


Figure S12. a) Maximum power point measurements in ambient air of FAPbI_3_ devices 1 M, 0.7 M and 0.5 M deposited in air and in glovebox b) Ambient air shelf life of FAPbI_3_ devices 1 M, 0.7 M and 0.5 M deposited in air and in glovebox

1. Targhi FF, Jalili YS, Kanjouri F. MAPbI_3_ and FAPbI_3_ perovskites as solar cells: Case study on structural, electrical and optical properties. *Results Phys*. 2018;10:616-627. doi:10.1016/j.rinp.2018.07.007
